# SARS-CoV-2 genomic epidemiology: data and sequencing infrastructure

**DOI:** 10.2217/fmb-2021-0207

**Published:** 2022-07-28

**Authors:** Georgi Merhi, Jad Koweyes, Tamara Salloum, Charbel Al Khoury, Siwar Haidar, Sima Tokajian

**Affiliations:** ^1^Department of Natural Sciences, School of Arts & Sciences, Lebanese American University, Byblos, Lebanon

**Keywords:** low- and middle-income countries, molecular epidemiology, next-generation sequencing platforms, SARS-CoV-2, sequencing

## Abstract

**Background:** Genomic surveillance of SARS-CoV-2 is critical in monitoring viral lineages. Available data reveal a significant gap between low- and middle-income countries and the rest of the world. **Methods:** The SARS-CoV-2 sequencing costs using the Oxford Nanopore MinION device and hardware prices for data computation in Lebanon were estimated and compared with those in developed countries. SARS-CoV-2 genomes deposited on the Global Initiative on Sharing All Influenza Data per 1000 COVID-19 cases were determined per country. **Results:** Sequencing costs in Lebanon were significantly higher compared with those in developed countries. Low- and middle-income countries showed limited sequencing capabilities linked to the lack of support, high prices, long delivery delays and limited availability of trained personnel. **Conclusion:** The authors recommend the mobilization of funds to develop whole-genome sequencing-based surveillance platforms and the implementation of genomic epidemiology to better identify and track outbreaks, leading to appropriate and mindful interventions.

## Plain language summary

Lebanon and other low- and middle-income countries have limited sequencing capabilities. Sequencing costs using MinION in Lebanon were higher than the approximate sequencing costs in developed countries. The challenges faced by low- and middle-income countries include lack of support, few established sequencing facilities, high prices, long delivery delays and the limited availability of trained personnel. There is a need to focus on the development of whole-genome sequencing-based surveillance platforms and the implementation of genomic epidemiology to improve sequencing efforts in many resource-limited settings and to contain and prevent future pandemic-level outbreaks.

## Tweetable abstract

Sequencing costs of #SARS-CoV-2 in Lebanon are higher than those in developed countries. #LMICs have limited #sequencing capabilities. Whole-genome sequencing-based surveillance platforms and the implementation of genomic epidemiology could improve sequencing efforts.

SARS-CoV-2 emerged in 2019. The uncontrolled transmission of SARS-CoV-2 is facilitating and providing the conditions for significant virus evolution, which is raising a widespread concern. Two years later and as of 15 February 2022, more than 8 million SARS-CoV-2 complete genome sequences have been submitted on the Global Initiative on Sharing All Influenza Data (https://www.gisaid.org/) [1], allowing for the rapid sharing, analysis and tracking of SARS-CoV-2 through genetic sequence analysis linked to clinical and epidemiological data, along with the geographical distribution and virus spread [2].

Genomic surveillance of SARS-CoV-2 is key in monitoring and tracking viral lineages circulating in each country. Sequencing and public sharing of SARS-CoV-2 genome data are crucial in tracking the evolution of the virus, allowing the identification of mutations and, in turn, tracking the emergence of new variants [3]. New variants could show altered behavior, transmissibility and disease severity and could impact the effectiveness of treatment and vaccines [4].

Nevertheless, the genome data distribution shows a large gap between low- and middle-income countries (based on World Bank classification, 2021 [5]) and the rest of the world [6]. The authors estimated the sequencing costs per SARS-CoV-2 genome using the Oxford Nanopore MinION platform in Lebanon and compared it with sequencing costs in developed countries. SARS-CoV-2 genome representation/country was also evaluated to demonstrate inequities between low- and middle-income versus developed countries.

## Methods

Sequencing costs per SARS-CoV-2 genome using the Oxford Nanopore MinION platform in Lebanon were estimated and compared with those in developed countries. The authors' estimates were based on the cost of reagents and materials recommended by the ARTIC SARS-CoV-2 Nanopore sequencing protocol V.1. The protocol involves cDNA preparation, amplification, cleanup, library preparation and MinION sequencing [7]. To account for cost differences linked to shipping costs and supplier profit margins, an analogy-based cost estimation approach was used, which accounted for a 30% price increase per sequenced genome.

The authors extracted the total number of COVID-19 cases reported in each country from the World Health Organization's website (https://covid19.who.int/) and collected SARS-CoV-2 sequence data along with metadata, including the reporting country from the Global Initiative on Sharing All Influenza Data database (up to 15 February 2022) [1]. Only complete genomes were included in the analysis. The number of sequenced genomes per country was further confirmed using the CoV-Spectrum tool [8]. The genomes generated per 1000 cases metric was calculated by computing the ratio of a cumulative number of genomes to the cumulative number of positive COVID-19 cases, and multiplying the value by 10^3^, for each listed country (Supplementary Table 1). Maps were generated with MS Excel (2019 version). Datasets were assigned using the geography data type input and maps were consequently generated via insert figure function [9].

## Results

**Figure 1.**
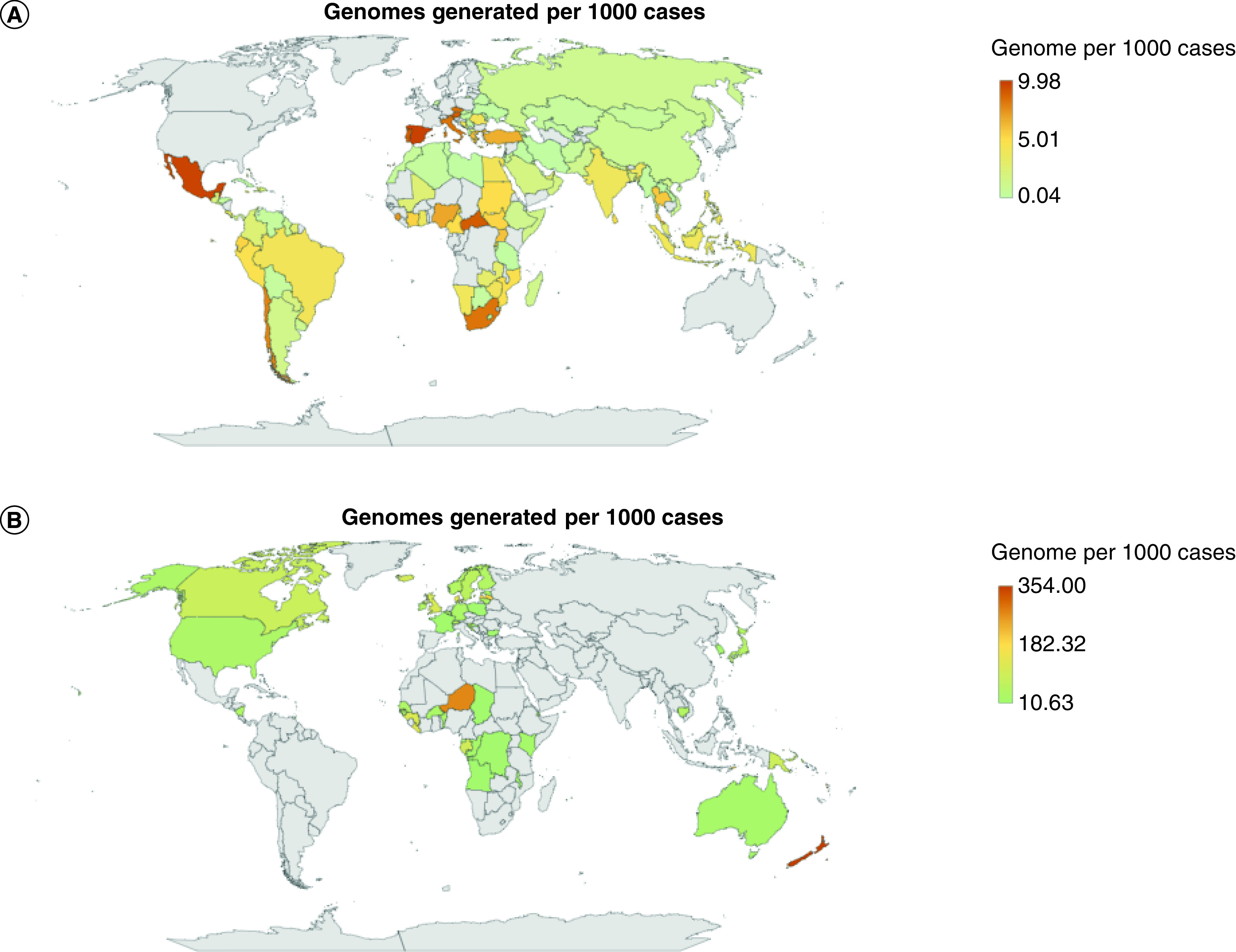
Genomes generated per 1000 positive cases per country (countries in white shown in panels [A] and [B] have no data). **(A)** Genomes generated per 1000 positive cases for countries in the range of 0.04–9.98 genomes. **(B)** Genomes generated per 1000 positive cases for countries in the range of 10.63–354 genomes.

Out of the 82 countries listed as low- and middle-income 85.4% (n = 70) had SARS-CoV-2-derived genome data deposited on the Global Initiative on Sharing All Influenza Data, with 76.8% (n = 63) having less than 1000 cumulative published genomes (Supplementary Table 2) [2]. The countries with the highest SARS-CoV-2 generated genome data were the USA (n = 2,606,331), UK (n = 2,064,939) and Germany (n = 399,167) and represented 33.86, 112.80 and 32.14 sequenced SARS-CoV-2 genomes per 1000 cases, respectively. The lowest numbers of deposited SARS-CoV-2 genomes were from Laos (n = 5), Tanzania (n = 3) and Palau (n = 2): 0.04, 0.09 and 0.64 sequenced SARS-CoV-2 genomes per 1000 cases, respectively (until 15 February 2022; data on the Global Initiative on Sharing All Influenza Data; Figure 1A & B & Supplementary Table 1).

**Figure 2.**
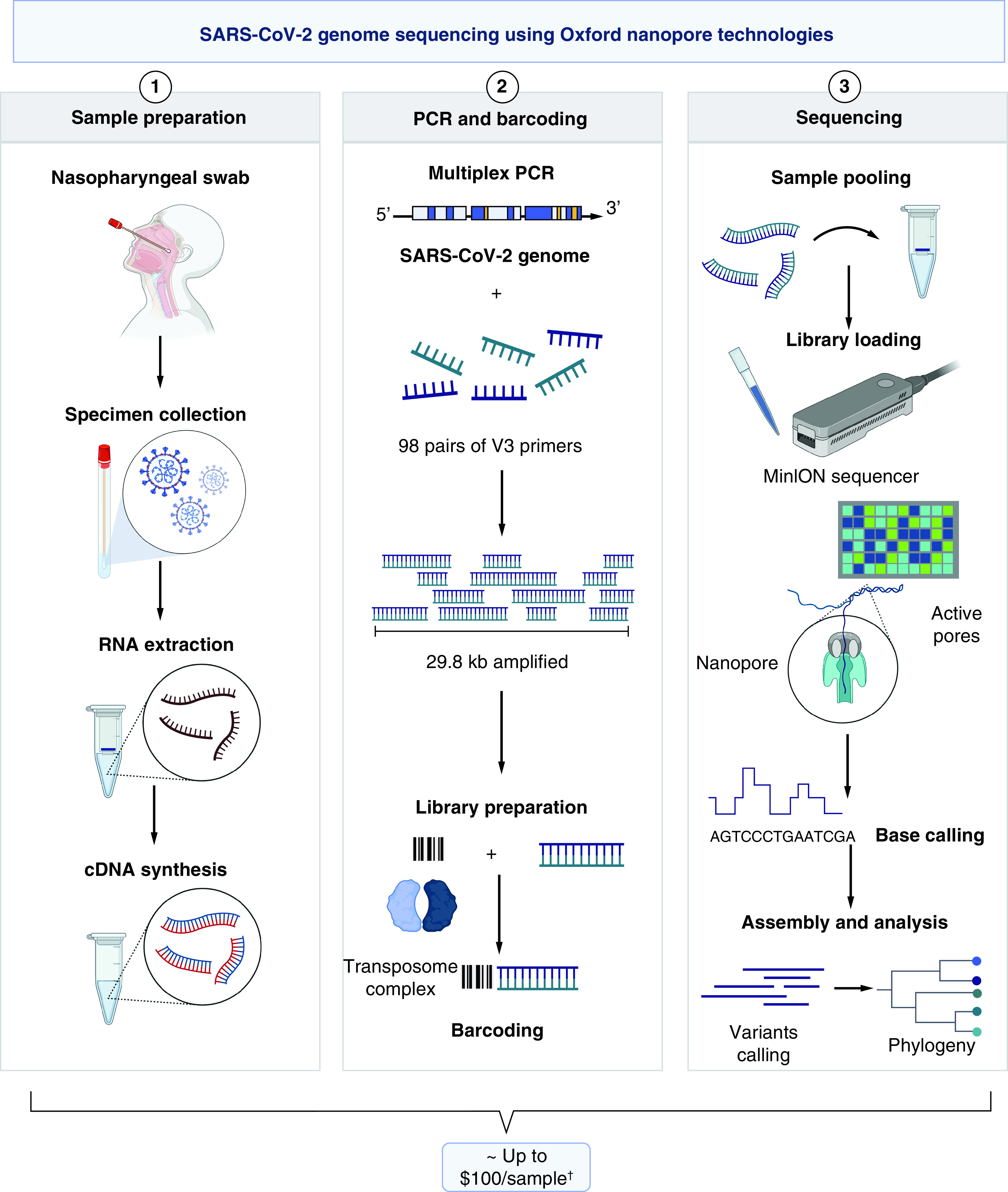
Workflow of SARS-CoV-2 sequencing using Oxford Nanopore Technologies. Price estimates were calculated based on international reagent prices plus 30% to account for shipping and local supplier profit. ^†^Based on price estimates of needed items per sample with a 30% added cost to account for local supplier profit margins and shipping costs. Image created with BioRender.com.

The cost of Nanopore sequencing per genome, following the ARTIC SARS-CoV-2 protocol, ranges between £33.42 ($44.24) and £55 ($74.46) [10,11] plus roughly 30% for shipment and profit margin, and so the estimated cost would fall between £43.44 ($57.51) and £71.5 ($96.79; Figure 2).

## Discussion

The observed differences in the number of deposited genomes could be attributed to the lack or unequal mobilization of funds [6]. The National Health Service, public health agencies, the Wellcome Sanger Institute and over 12 academic partners forming the COVID-19 Genomics UK consortium received more than £30 million to ensure large-scale and rapid SARS-CoV-2 sequencing (https://www.cogconsortium.uk/). Genome sequencing facilities in Low- and middle-income countries, in contrast, remain scarce which is often associated with limited resources being dedicated, if any, for teaching and research and development (R&D) [2,6].

**Figure 3.**
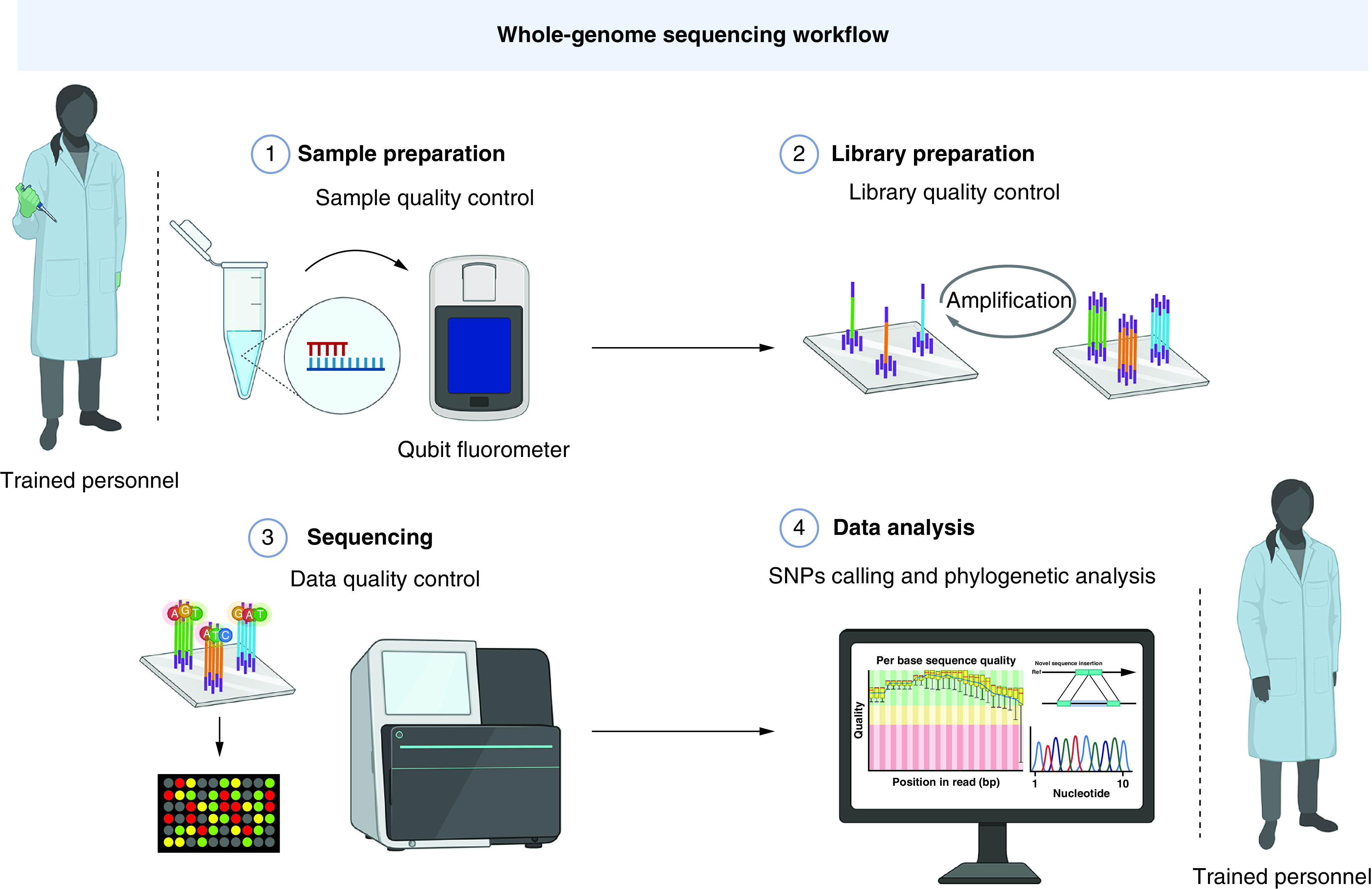
Summary of the main components of a next-generation sequencing facility and the standard next-generation sequencing workflow. Image created with BioRender.com.

Despite the remarkable increase in speed and the decrease in the cost of establishing genome sequencing facilities, especially with the emergence of next-generation sequencing platforms [12], covering the incurred costs remains challenging. The cost of establishing and running a next-generation sequencing facility in developed countries could range between $80,000 and $700,000 (Figure 3). The most expensive next-generation sequencing sequencer, the HiSeq 4000 (Illumina), costs £474,373, with an annual maintenance cost of £55,641 [13]. The estimated cost of exome sequencing could be in the range of £382 ($555) to £3592 ($5169), while it is £1312 ($1906) to £17,243 ($24,810) for human genome sequencing [14]. Low- and middle-income countries additionally need to account for shipping, customs and local supplier profit margins [6,15].

Oxford Nanopore Technologies provides a more accessible and affordable solution for rapid SARS-CoV-2 sequencing [14], although with an error rate of around 14% [16]. Sequencing using the MinION and following the ARTIC-based protocol (https://artic.network/ncov-2019) would cost around £43.44 ($57.51) to £71.5 ($96.79) per genome (Figure 2), which is significantly more than the cost/genome ($35.88) using the same platform in the USA [17] while being consistent with that reported in other low- and middle-income countries ($110 per genome in Uganda) [18].

Another challenge is establishing and having the much needed computational infrastructure to cope with the exponential increase in the demand for data storage, processing and analysis [19]. Although the MinION ensures low cost with minimal necessary hardware [13], with the average retail cost being $2000–$4000, the turnover of high accuracy base calling can be relatively slow, at 4.4 kb/s if it is only relying on central processing unit computation (https://nanoporetech.com/community/lab-it-requirements). Graphics processing units, however, are viable options for high-performance computing and increased scalability [20]. Utilizing graphics processing unit computation to generate sequencing data from the MinION device would improve the high accuracy base calling speed (22,000 bases/s) but would also increase the average retail price (minimum 8 GB of graphics processing unit memory being recommended by Oxford Nanopore Technologies [https://nanoporetech.com/community/lab-it-requirements]).

In addition to the instrumentation expenses, there are considerations around time and personnel expenditures associated with employing dedicated full-time employees to follow up and maintain the continuity [21].

In Lebanon, as of the date of writing this manuscript, 1192 SARS-CoV-2 genomes were deposited on the Global Initiative on Sharing All Influenza Data, representing 1.17 of the overall sequenced genomes per 1000 cases. COVID-19 allocated funds were limited due to the experienced socioeconomic instability [22] and the lack of stimulus packages to better equip hospitals [23].

## Conclusion

Although sequencing efforts in some low- and middle-income countries, including Vietnam, Gambia, Egypt, Congo and Lebanon, have been stimulated by the pandemic, the unequal genome sequence coverage/country has persisted throughout the pandemic. These inequities could be primarily due to the presence of few established sequencing facilities, the lack of funds and a scarcity of skilled personnel. We recommend the mobilization of funds to support capacity building in bioinformatics and the adoption of modern sequencing technologies in resource-limited settings.

## Future perspective

Whole-genome sequencing has added a level of precision and led to the development of a faster and better response to infectious diseases. Creating affordable genomic services to drive high-level science should encompass investing in other fields, and particularly in bioinformatics and information technology, to improve real-time surveillance, outbreak investigations and epidemic preparedness. Although sequencing efforts in some low- and middle-income countries have been stimulated by the pandemic, the caveat of unequal genome data representation will remain unless global efforts are redirected toward the mobilization of funds to support capacity building in bioinformatics and the adoption of modern sequencing technologies in resource-limited settings. Developing and scaling up sequencing capacities in low- and middle-income countries is essential for genomic surveillance and worldwide monitoring of SARS-CoV-2 variants and other pathogens. Pathogen surveillance and tracking variants can help in expediting and developing a more effective and timely response. The development of whole-genome sequencing-based surveillance platforms and the implementation of genomic epidemiology will allow health systems to better identify and track outbreaks, leading to appropriate and mindful interventions.

**Summary points**

- Whole-genome sequencing has added a level of precision and led to the development of a faster and better response to infectious diseases.
- The development of whole-genome sequencing-based surveillance platforms and the implementation of genomic epidemiology will allow health systems to better identify and track outbreaks, leading to appropriate and mindful interventions.
- Sequencing efforts in some low- and middle-income countries have been stimulated by the pandemic, but the caveat of unequal genome data representation is still evident.
- Inequities are associated with few established sequencing facilities, high costs, long delivery delays and the limited availability of trained personnel.
- Developing and scaling up sequencing capacities in low- and middle-income countries is essential for genomic surveillance and worldwide monitoring of SARS-CoV-2 variants and other pathogens.
- Resources are urgently needed to establish better-equipped sequencing facilities, strengthen the infrastructure and support training and development programs in low- and middle-income countries.
